# Navigating resistance on digital platforms: delivery and transportation labor in Türkiye

**DOI:** 10.3389/fsoc.2024.1456617

**Published:** 2024-12-11

**Authors:** Batuhan Ersöz, Altan Başaran

**Affiliations:** ^1^Department of Social Work, Faculty of Health Sciences, Tokat Gaziosmanpaşa University, Tokat, Türkiye; ^2^Department of Labor Economics and Industrial Relations, Faculty of Economics, Marmara University, Istanbul, Türkiye

**Keywords:** platform work, labor struggle, informal resistance, platformization, digital platforms

## Abstract

Digital platforms are transforming the world of work. However, platforms operating in similar fields of activity encounter varying mechanisms of opposition, as a result of different degrees of professional institutionalization and their relations with the state. This study examines the diversified labor/capital struggle processes on platforms operating at different points of urban mobility in Istanbul and makes an evaluation between delivery and transportation platforms. Therefore, the actions of workers against digital platforms, news reports and public statements of relevant actors in both fields were systematically analyzed. As a result of the study, it is seen that the labor struggle in delivery services includes demands for the regulation of work in parallel with traditional working class reflexes. On the other hand, the resistance in the urban transport platforms is formed in the center of the rent and turns into an institutional struggle as a result of the public activity of the actors. Thus, the inter-class struggle in delivery services operating on digital platforms transforms into an intra-class rent-sharing struggle in urban transport.

## 1 Introduction: platform work through the lens of labor process and property relations

Platform-based work has become a global phenomenon, covering a wide range of workers due to its success in connecting supply and demand (OECD, 2019). The rapidly evolving new economy has led to the swift development of the gig work market, increasing the number of non-standard jobs. For example, the number of platform workers in the European Union, estimated at 28 million, is expected to reach 43 million by 2025 (European Commission, 2021). Unlike the standard process in traditional workplaces, platform workers log on to the platform systems and accept the job/task or project requested on the system to be completed within a specified time period. Moreover, platforms that can operate in a wide spectrum close the trust gap that spatial dispersion would create in interpersonal relations. The intermediary nature of platforms eliminates trust issues between service providers and consumers (Berg et al., 2020). Thus, platforms often act as a bridge between those who demand the service and those who provide it, acting as an important intermediary between the parties.

The platform economy characterizes workers as “independent entrepreneurs / contractors” and envisions a tripartite structure in which independent workers and customer demands instantly intersect through applications (Florisson and Mandl, 2018). This process creates a controversial status for the workers in the platform economy (De Stefano, 2016). The activities of the platforms, which are shaped by big data and algorithms, make algorithmic management an important mechanism of organizational management and labor control (Gandini, 2019; Möhlmann et al., 2021; Jooss et al., 2022; Caza et al., 2022; Parent-Rocheleau and Parker, 2022; Huang, 2023). This situation creates an important field of discussion for the labor process and offers major opportunities for understanding the dynamics of working life, which is being reorganized depending on digital platforms. Digital platforms weaken the power of workers in employment relations, leading to the equalization of wages at the lower end of the scale (Veen et al., 2020; Huang, 2023). In addition, although traditional employment relations have protection mechanisms for workers, working through platforms individualizes the risks associated with working life (Webster, 2016; Duggan et al., 2020; Shanahan and Smith, 2021; Shvetsova, 2022).

The platform ecosystem also prevents workers from accessing their fundamental right to organize a union. The organizing practices of trade unions are not effective for gig workers, who are a heterogeneous and individual group of workers (Lee, 2023) in terms of the political and legal dimensions of their conditions. The need to regulate the political and legal aspects of this situation makes the relations between gig workers and trade unions uncertain (Kresal, 2022). The fact that platforms bring gig workers together in a centralized structure, even for a certain period of time, creates a paradox in terms of organizing these workers (Naidu, 2022). However, the digital possibilities that enable platforms to access global markets are creating a communication network for platform workers. Workers’ efforts to find solutions to their problems in different ways through global/local networks support a movement towards new forms of organization (Eurofound, 2018; Wood et al., 2018; Yenisey, 2022; Schou and Bucher, 2023).

In this context, three different forms of organization are encountered in platform struggles at the global level. Workers on platforms can take part in different online communication processes such as online activities. They can form unions and organizations, or existing unions can support labor struggles on platforms (Yenisey, 2022). Platform workers contribute to the development of new and informal models by transforming organizational practices into a structure suitable for the digital age (Roşioru, 2022; Però and Downey, 2024). Bonini and Treré (2024) point out that resistance practices on platforms include individual practices such as operating on more than one platform, having more than one account on a platform, acting outside platform directives, and collective practices such as solidarity log-outs and coordinated cancellation of orders, emphasizing that resistance processes are not only directly against algorithms, but also through algorithms.

This study aims to evaluate the digital platform relations located in different sectors and to explain the differentiated nature of the resistances seen against digital platforms in terms of urban mobility. The delivery and urban mobility platforms, which constitute the main object of review in this study, provide a significant example of different labor struggles. Since 2015, there has been a wide repertoire of labor actions on these platforms, ranging from mass log-offs to public protests and strikes on a global scale (Maccarrone and Tassinari, 2023). In fact, the Uber driver protests in the U.S., which spread across different states, achieved different forms of action and gains, as well as the support of traditional unions and differentiated labor groups (Collier et al., 2017). The global nature of platforms can enable global actions in terms of labor movements. An important example of this is the international strikes against urban mobility platforms Uber and Lyft in 2019, ranging from the United Kingdom and the United States to Brazil and Australia, with various traditional unions supporting these actions (Chesta, 2023). Moreover, as seen in the case of Spain, the activities of platform workers organized through associations or professional organizations can have similarities to the activities of traditional trade unions (Fernandez and Barreiro, 2020). This organizational model also indicates the dominant form of organization for platform workers (Stuart et al., 2023). Traditional unions differ in how they have approached supporting platform-based struggles. Unions’ influence in platform labor struggles can be positioned in the tension between responding to the needs of existing members and reaching out to potential members (Castel-Branco et al., 2023). In some instances, trade unions may adopt a strategy that prioritizes the protection of their existing members’ rights (Atzeni, 2023). Occupational organizations may have a similar approach to the unions’ approach to labor struggles on platforms, and may have an attitude of activism that aims to prevent the competition created by platforms and protect the interests of their members (Collier et al., 2017).

The effectiveness of digital platforms is not homogeneous across countries and economies around the world. Many factors, such as the structure of a country’s labor markets, its infrastructure, institutionalization level and market mechanism, impact the efficiency of platforms and labor activities (Fairwork, 2021; OECD, 2023). These differences are clearly seen in the Digital Platform Economy Index, where digital platform effectiveness is evaluated on the country level. With the data of 2020, the United States, the United Kingdom and the Netherlands ranked in the first three places among 116 countries, while Madagascar, Burundi and Ethiopia ranked in the last three. Meanwhile, Türkiye ranks 49th with a score of 32.3 (Szerb et al., 2022). Furthermore, digital platforms have an impact not only on the labor that works within them, but also on professional actors working in similar professional fields. This situation leads to struggles against the operations and activities of the platforms. The demands of the actors may differ in a wide range from the level of institutionalization of the professional fields to the political effectiveness of the actors. Likewise, in Türkiye, there are platforms such as “Getir,” “Vigo,” “Yemeksepeti,” “Trendyol Go” in delivery services, “Uber,” “Martı” in transportation services, and “Armut,” which operates in a wide range of activities from home renovation to maintenance services (Öz, 2023; Uysal, 2023). These platforms can either internationalize after their local emergence or have a direct international character. This transitivity creates a multi-organizational and multi-actor regulatory and operational process related to the activities of the platforms.

This study evaluates the different dimensions of couriers’ and urban transport actors’ struggles/resistances against spatially organized digital platforms in Istanbul. This exploratory qualitative study aims to analyze why actors in two different professional fields develop different forms of struggle against digital platforms based on their class positions. In this study, the structure of the struggles of couriers working in delivery services and traditional taxis in urban transport services against digital platforms in Istanbul are examined in the context of the possibilities offered by labor process theory and proletarianization.

In understanding the different forms of struggle against the platforms, the conceptual framework offered by labor process theory provides a significant framework for the working conditions and employment relations of couriers. On the other hand, the struggle of traditional taxi actors, which represents a different class position, gains ground on the basis of proletarianization and property relations. The labor process mainly examines the transformation of labor and the workplace relations, differentiated control and consent mechanisms on the basis of the production process (Braverman, 1974; Burawoy, 1979, 1985; Thompson, 1990). As Braverman (1974) emphasises, the production process is constantly reorganised around capital accumulation. As labor becomes increasingly detached from the knowledge of production, the control and supervision shifts directly to the employers. This approach focuses on a dual control mechanism and is therefore criticized for being a linear approach and not taking into account the different stratifications within labor (Littler, 1990; Thompson, 1990). On the other hand, Burawoy (1979, 1985) focuses on differentiated control mechanisms in terms of the labor process and includes the mechanisms of consent generation through various production organizations in the framework of the discussion. Thus, it focuses on the reconstitution of the capitalist production process within the scope of diversified control mechanisms. According to this analysis, the capitalist labor process has three main dynamics: *job design and division of labor*, *control structure* in a broad framework from monitoring processes to rewarding strategies and *employment relations* that includes market structure, property relations and the state. Therefore, the labor process refers to both property relations and general control mechanisms (Littler, 1990). The development of theories on the labor process demonstrates a dynamic structure. Different areas of research are integrated into the development processes depending on the changing relations of production. In this context, Thompson and Newsome (2004), who examine labor process theories through four waves, note that the fourth wave in particular emphasizes global production chains and new production relations in which risks are individualized. This approach is especially important regarding the characteristics of digital platforms that shape the labor process.

However, the impact of digital platforms is not only observed between labor and capital in the context of the reorganization of work. Digital platforms also constitute an important field of competition for traditional actors and forms of work. This diversifies the processes of struggle within similar digital platforms and differentiates the struggles between “labor and capital” and the struggles between “different layers of capital.” In this context, the relations of small-scale producer forms with digital platforms are situated in a significant point, and the change of property relations and social positions should be analyzed on this context. This situation also leads to the discussion of the occupational rent that small producers have owned based on their property. Rent is decisive in terms of social class positions formed within the framework of certain property relations (Marx, 1990). However, especially small producers are in danger of dispossession and proletarianization on the basis of capitalist competition (Savran, 2014). The fact that the position of small producers in existing production relations is based on permanent property rights linked to rent makes these class positions more fragile. Small producers, on the other hand, organize themselves through associations such as professional associations to protect their rent-based advantages and intervene in competition processes by institutionalizing (Parkin, 1979).

In regard to this subject, the existing literature is initially examined, and then the news of actions, statements of policymakers, professional actors, and unions are analyzed in reference to both research areas. Finally, the reasons for the differentiation of struggles against platforms in traditional taxi services and delivery services are discussed in terms of employment status, class positions and property relations. This leads to the differentiation of the forms of struggle developed by different class positions against digital labor platforms. Therefore, the actions and struggles related to digital platforms, which can be traced through two examples in Istanbul, constitute an important example in terms of observing the struggles developed in different property relations and class positions.

## 2 Method and limitations

This study descriptively examines two different cases of struggles on digital platforms (“Yemeksepeti” and “Trendyol” platforms for *delivery services* and Uber platform for *urban transportation* services) in Istanbul. Exploratory qualitative research focuses on a group or individual’s understanding of a social problem, providing meaning and perspectives on actions and events (Creswell and Creswell, 2017; Maxwell, 2012). Thus, new areas of inquiry are being developed (Patton, 2014) that provide an understanding and exploration of the unique dimensions of concepts as social phenomena (Maxwell, 2012; Merriam and Tisdell, 2015). The study reviewed secondary data, public and political statements, and news regarding the struggles. As the analysis period of the study, the years 2018–2023 were taken into consideration, as the years in which the struggles and regulations in the relevant field were widely observed.

For both cases, in addition to the relevant literature, the public news and public statements made by the relevant actors are taken into consideration. The electronic news archives of various public media platforms were used to access the news and statements by actors and institutions. During the search process, certain keywords such as “courier protests,” “taxi resistance,” “Uber statements,” etc. were used to retrieve news based on the analysis period of the study. However, the news and the statements of actors and press releases used in the study do not represent a quantitative categorization, but provide a reference to the relevant facts within the scope of this review. Moreover, a wide range of statements from different professional organizations, especially from relevant public figures, as well as company statements were examined. While evaluating the processes of struggle in the delivery services, the approaches of the two major trade unions in the relevant sector, Nakliyat-İş (Revolutionary Land, Air and Rail Transport Workers Union of Türkiye) and TÜMTİS (All Transport Workers’ Union), as well as the news portals of the couriers in particular, were also included. However, since urban transportation is also a public service, it exhibits a more complex and multi-actor structure compared to delivery services. Therefore, in addition to the statements of the Istanbul Chamber of Taxi Drivers (İTEO) and Istanbul Taxi Drivers’ Association, which are important professional organizations regarding traditional taxi services, the evaluations of local government actors were also taken into consideration. For this reason, the study also includes the statements of the then Mayor and the mayoral candidates of the two major political parties of the period.

In this direction, first, the labor relations of couriers working on different digital platforms are focused and class struggles in this field are addressed. Furthermore, to comprehend the organizational processes, labor processes were analyzed with particular attention focused on the union approaches within this framework. Then, the emerging competition in urban transportation due to digital platforms and its effects on existing professional actors are emphasized. The conflicts between traditional taxis and Uber in Istanbul serve as the focus within this context. The evaluation concentrated on the institutional and informal struggles of traditional actors in competition with digital platforms. In addition, intervention strategies against digital platforms were reviewed based on the statements of professional actors and policy makers. It should be noted, however, that the study is not without limitations. First, the study includes the demands of relevant actors, as documented in the public sphere. Secondly, the case of Istanbul constitutes a natural limitation due to its population density and the economic efficiency and intensity of the platform activities. Finally, the platforms examined were chosen due to the fact that they were the platforms where the most intense debates and labor movements were observed in the relevant period.

## 3 Digital labor platforms and delivery/transport workers in Istanbul: differentiating forms of resistance

The increasing mobility of people and goods on a global scale brings new forms and methods of mobility into the agenda, based on flexibility and more intensive use of digital tools (Krastev, 2017). These developments are directly parallel to the growth in the platform economy, and digitalization creates new possibilities in terms of mobility. It is also observed that digital platforms have the ability to combine and diversify services with different functions in the urban space. For example, the firm “Uber” can provide both a service for urban transportation and also offers business models for on demand food delivery service. Discussions in this area usually focus on the labor process on platforms (Gandini, 2019), labor relations (Kullmann, 2022; Howson et al., 2022) and new organization dimensions (Joyce et al., 2023). However, platforms do not only change labor relations in the production process, but they may also confront traditional actors in some professional areas as well.

In order to gain insight into the processes of institutionalization in the field of delivery and transportation in Türkiye, it is useful to provide a brief overview of these processes. In Türkiye, delivery services are a relatively new field on the line of late development of capitalism. Especially since the 1980s, this service area has expanded in parallel with the economic transformation Türkiye has experienced (Kıdak, 2021; Kaya et al., 2022). This expansion process can also be traced in terms of employee organizations, with 12 trade unions operating within the relevant sector. It is stated that the existence of different unions in the relevant field also results in union competition (Kıdak, 2021). Moreover, the gradual increase in the number of couriers working independently through platforms, which is a common phenomenon across platforms, brings about the transformation of the organization of labor in this sector. Urban transportation, on the other hand, is more complex in terms of both institutional and organizational structure, and the late capitalist nature of urban development in Türkiye leads to the predominance of informal elements in the organization of urban services (Tekeli et al., 1976). In examining the historical development of urban transportation, it becomes evident that a multi-actor structure has emerged, comprising both private and public actors. In the case of Istanbul, taxis operate as independent operators depending on the regulations of the local government. The structure of taxi operation based on private ownership does not indicate a large-scale corporatization, and urban transportation is provided through individual license ownership. Thus, the urban transportation service provided by taxis is under the control of the local government, but on an individual basis. The local government controls the number of taxi licenses. While the population is constantly increasing, the number of taxi licenses does not increase at the same rate or remains fixed, leading to the renting of licenses. In addition, taxi license holders have historically struggled to prevent competition by limiting new licenses (Altınoklu, 2023; Tekeli, 2010). Moreover, taxi drivers can be direct license holders, employee-drivers working under the license holder, or independent drivers who rent the license (Ergün et al., 2020). This causes the emergence of a complex multi-actor structure in taxi transportation, distinct from delivery services. Therefore, the two service areas must be elaborated separately.

### 3.1 Struggle within the digital platforms: case of delivery workers in Istanbul

The employment structure of on-demand delivery services, which have been growing in recent years, is similar to the self-employment process described above, and actors in this field generally work as independent contractors. The logic of outsourcing, which is shaped by costs and based on flexibility in the capital accumulation process, also becomes central to the organization of work in delivery services offered by (Goods et al., 2019). Controlling labor supply at the spatial level is particularly important for professions that require continuous activity in urban space, such as delivery services, and through digital platforms, idle labor at the spatial level can be operationalized. In this context, the issues raised regarding self-employment are also present in the case of Türkiye.

Couriers operating within digital platforms have been encountering a diversified repertoire of struggles and organizing processes since the early 2020s to have their specific demands met by the platforms (Abilio et al., 2021; Woodcock and Cant, 2022). For example, Stuart et al. (2023), who examined labor unrest in 19 firms and 95 countries between 2017 and 2020, found that the highest number of incidents occurred in Europe with 51%, followed by Asia with 25% and South Africa with 17%. There are also significant differences between the effectiveness of traditional unions and informal groups in these struggles on a global level. The struggles seen on the platforms are mostly through informal organizations and include direct struggles of workers (Stuart et al., 2023). Within the framework of the study, the actions of couriers operating in “Trendyol” and “Yemeksepeti,” which are important examples of delivery services among digital platforms in Türkiye, are analyzed. There are similarities to the global field in both the beginning of the protests and the workers’ demands. In this regard, it is important to elaborate the labor struggles that have developed on the basis of both platforms.

These platforms started their operations locally, but later acquired a global character. “Yemeksepeti” was sold to Germany-based “Delivery Hero” in 2015, while 86% of the “Trendyol” platform was acquired by China-based “Alibaba Express” (Öz, 2023). Both platforms have been confronted with labor protests that have been on the public agenda since the beginning of 2022. A series of demands regarding the organization of work, in particular the delivery payments, were made to the platforms, while collective “ignition shutdowns” and slowdown strikes were carried out (Evrensel, 2022; Serdaroğlu, 2022). During the protests, which were also supported by trade unions, support campaigns were organized on social media with various boycott calls to raise awareness (Coşan, 2022). As a first example the actions of the self-employed couriers working with Trendyol company are evaluated. At the beginning of 2022, an 11% increase was put on the agenda by the platform, and in response, the couriers organized a three-day protest called the “Legendary Resistance Days “, in reference to the platform’s advertising slogan. As a result of these protests, a 38.8% increase was accepted by the platform (Kurye Haber, 2022).

Furthermore, couriers working for Trendyol were involved in a similar action process at the beginning of 2023, stopping work and staging protests in front of the company’s headquarters, demanding improvements in both income and working conditions (Birgün, 2023). The demands of couriers in this area are particularly meaningful in terms of the control that platforms have over the labor process. The 22-item list of demands created by couriers included improvements in income, as well as limiting transportation distances, regulating peak hours and limiting algorithmic control processes. There were also demands for social protection, including the extension of recruitment and employment periods, the provision of health insurance coverage by the platform, and the prevention of dismissals of couriers due to the stated demands (Duvar, 2023). As a result of the actions, the platform management made a statement and responded positively to the increase in income demands and stated that demands regarding administrative processes would also be evaluated (Cumhuriyet, 2023).

The action process of the couriers working on the “Yemeksepeti” platform in 2022, which lasted for more than a month and included statements and actions at different levels, is another example of the actions of workers in delivery services connected to digital platforms. It is noted that the fact that those working on this platform consist of both paid employees and self-employed couriers has led to the formation of a much more complex labor movement (Öz, 2023). Therefore, the most important demands in these actions were, in addition to the improvement of income, the demands for the right to organize and the end of the practice of “self-employed” couriers. As a matter of fact, the firm also changed the “branch of activity” in order to prevent unions from organizing, and the union membership of 2000 union members was revoked (Öz, 2023). In the statements made in front of the headquarters of the platform during the work stoppage, there were active calls for support and boycott from relevant unions such as Nakliyat-İş (Revolutionary Land, Air and Rail Transport Workers Union of Türkiye) and TÜMTİS (All Transport Workers Union), and demands for the right to organize were expressed (Kurye Haber, 2022).

During this process, in addition to actions such as slowdown strikes and press releases, a strong social media solidarity network was created with the call of trade unions (Coşan, 2022). The calls for a boycott of the platform also resonated with the public, with artists and journalists sending messages of solidarity with Yemeksepeti couriers through social media channels. At the same time, the Nakliyat-İş stated that after the boycott call, there was a 70% drop in orders from the platform (Nakliyat-İş, 2022). However, it is observed that the labor movements and boycott calls, which gained public visibility in the early periods, were insufficient in terms of sustainability in the following periods and gradually faded away without any gains (Kurye Haber, 2022). It is also stated that the platform transformed its bureaucratic internal structure during the action process and tried to prevent existing labor movements by changing the disciplinary regulation (Serdaroğlu, 2022).

In this context, a differentiated process of struggle emerges for delivery workers operating on digital platforms. In Türkiye, as evidenced by the examples in the global literature, the demands of platform workers encompass a wide range of areas, from the establishment of social protection mechanisms to the right to unionize, particularly the elimination of income insecurity. Their means of struggle include calls for work stoppages, slowdown strikes, and boycotts. The couriers’ demands regarding their working lives actually include a demand for the establishment of a norm parallel to other examples in terms of industrial relations (Joyce et al., 2023). The important area of discussion in both cases is the status of being an “independent contractor,” which structurally points to the outsourcing of capitalist development, and demands for recognition as “employee” occupy a central place.

The structure of labor formation, which transforms into a grey area in terms of delivery services through platforms in urban space, can produce heterogeneous results in terms of different digital platforms that constitute mobility in urban space. The effectiveness of platforms can be limited, especially depending on the strength of local actors. Therefore, the next sub-heading will evaluate the nature of competition between traditional taxi drivers and the Uber platform. In order to understand the different forms of struggle, the focus will be on the struggles in formal and informal relations and on the consequences of the approaches of the different public actors.

### 3.2 Occupational segregation, rent and platform work: traditional taxi resistance against digital platforms in Istanbul

Urban transportation has also experienced significant changes as a result of the effectiveness of digital platforms. The changes in this area have had a significant impact in Istanbul, a city of approximately 16 million people, especially when it comes to taxis. In Istanbul, as in many other parts of the world, taxi licenses are granted to individuals and these licenses are embodied in the license plates of their vehicles (Yıldızgöz, 2018). Thus, a structure is observed in where a public service is provided by only private actors. However, in Istanbul, the Istanbul Metropolitan Municipality (İBB) has the authority in urban transport under the Metropolitan Municipality Law No. 5215, and since urban transport is also an important public service, all decisions related to transport are implemented through the municipality’s subdivisions related to transport. Taxi services, as a form of urban transportation, demonstrate the provision of a public service by private entities within a framework where individuals own the right to operate. The responsibility to regulate this service lies with the local government and its associated bodies. Although the number varies, there are approximately 20,000 taxis in Istanbul (İBB, 2023) and over 50,000 taxi drivers according to information in 2020 (Ersöz, 2022).

The most important regulatory sub-governmental body in this field is the Transport Coordination Centre (Ulaşım Koordinasyon Merkezi – UKOME), which is required to be established in Article 9 of the relevant law. The UKOME comprises representatives from local and central government, as well as transport-related actors, and its decisions are legally binding on all parties. However, with the amendment published in the Official Gazette No. 31044 dated 19 February 2020, the membership structure of UKOME was restructured to increase the weight of central government representatives. This has resulted in the central government having authority in some areas of regulation where the local government was previously decisive.^1^ In this context, as a digital platform providing urban transport services, Uber is directly subject to the relevant regulations.

The firm Uber, which began operations in 2009 and provides a very important example of the relation between urban mobility and digital platforms, has followed a rapid growth graph, reaching 8 million users and 160,000 drivers in 250 cities in 2014 (Wallsten, 2015). Uber’s activities directly raise debates on competition, taxation and working conditions (Pepić, 2018). In the context of this study, this has led to a central position in the debate about competition, given the negative impact of Uber’s activities on the traditional taxi drivers’ earnings (Berger et al., 2018). Competition in existing taxi services can also be seen in the restrictions imposed on taxi licenses in Istanbul. The population in Istanbul has increased by 117% since 1990, while the number of taxis has increased by only 5.7% (İBB, 2023).

In Türkiye, Uber’s effects have been observed since 2014. To the extent that Uber has substituted for traditional taxi transport in urban transport in Istanbul, it has caused a decline in both the potential income and the exchange value of taxi licenses directly on the market. Thus, the decline in potential income as a result of Uber’s activities problematizes both the income of drivers who provide direct labor in urban transportation and the rental income of small producers who are license holders. In fact, this situation can be easily observed in terms of the market value of taxi licenses in Istanbul. In 2018, the period when Uber’s activities and public visibility started to increase, the price of taxi licenses decreased by about 30% (T24, 2018). During this period, many different institutions and urban transport actors, especially professional associations, made statements about Uber’s activities in Istanbul. Taxi license holders, who are small-scale producers, demanded the cessation of Uber’s activities on the grounds that they were illegal.

The main problem with Uber’s transportation activities in Istanbul is that it is providing a similar service within a legal regime where only taxis are allowed to provide regular urban transportation. In Istanbul, Uber came on the agenda with the “UberXL” service, which provides service with luxury segment service vehicles. These vehicles operate in Istanbul with a D2 license. The D2 certificate for domestic non-scheduled transportation, which is mostly used in the tourism sector, allows for pre-invoiced and pre-contracted services. However, as a digital platform, Uber is positioned in a grey area by combining spatial mobilization with technological efficiency (İnci, 2018). In fact, it provides a pre-planned service similar to regular transportation without a proper transportation license. This situation points to the main problem with Uber and actually enables those who are not authorized to operate in this field to organize through platforms. As a matter of fact, for a similar urban transportation platform, the “BiTaksi” platform does not face such a problem as it organizes existing licensed taxis by mobilizing them into the digital space.

This situation has led the actors who work in the traditional taxi industry to develop a strong opposition to Uber. As a result of the increasing visibility of Uber’s activities in Istanbul, statements about Uber from many actors in urban transport became part of the agenda. In particular, various associations related to the taxi industry harshly criticized Uber’s activities. The then-president of the Istanbul Taxi Drivers’ Association even went further, declaring that Uber was operating illegally in Istanbul and accusing those who drove for Uber and used Uber of “treason” (Sputniknews Türkiye, 2018a).

The increasing level of strictness in the rhetoric of the opposition to Uber had repercussions on the ground, with threats and various acts of violence by taxi drivers targeting Uber drivers (Habertürk, 2018; Ulusal, 2018). This is an important example of informal organization and income control in urban transportation activities based on different levels of individual licensing. When individual licensing in urban transport is shaped by weak levels of institutionalization, potential income control is often achieved through informal actions and interpersonal relations, and informal actions may include acts of violence depending on the level of struggle (Fourie, 2005; Cervero and Golub, 2007; Spooner and Manga, 2019). It is observed that a similar process took place in Istanbul during Uber’s operations. It is noted that in the first quarter of 2018, when the controversy started, nearly 30 attacks were carried out against Uber drivers, and even in some incidents, taxi drivers attacked Uber drivers by demanding Uber service as customers (Sputniknews Türkiye, 2018b). However, these incidents have not only led to a public backlash against the already questioned taxis, but also increased the visibility of Uber. This was also mentioned by the former president of the Istanbul Chamber of Taxi Drivers (İTEO), who emphasized the need to improve the existing service through regulation (Eser, 2020).

Both the approaches of the formal professional actors of the sector and the opposition of the drivers directly through informal relations in the work process regarding Uber’s activities in Istanbul have been responded to by policy makers. Various statements have been made by local and central government authorities against Uber in the political arena. The statements of political actors in this field can be explained in terms of protecting local “voters “against Uber, an “external” actor. The then-mayor of Istanbul Metropolitan Municipality criticized the actions of taxis against Uber, stating that Uber was a “convenience” (Hürriyet, 2018a), but at the same time, during the local election process, statements such as “we are ready to stand by taxi drivers” were made. Similarly, the two candidates who received the highest number of votes in local elections during this period also issued statements opposing Uber (TRT Haber, 2019; Anadolu Agency, 2018; İBB, 2018). The President also made statements in support of taxi drivers regarding Uber’s activities during the same period (Hürriyet, 2018b).

In this process, the pressure of the professional actors yielded results in parallel with the consecutive statements of the political actors. First, Uber shut down its “UberXL” service, an alternative to taxis, on May 31, 2019 (Habertürk, 2019). Then, later in 2019, access to Uber was completely blocked by court order (Sputniknews Türkiye, 2019). After the ban period, there was an increase in both taxi license values (NTV, 2019) and taxi revenues directly related to urban transport (Diken, 2020). This shows that restrictions on direct competition had significant effects. However, the ban on access to Uber was removed at the end of 2020, but Uber’s activities could only be used for currently licensed taxis, in accordance with existing regulations (NTV, 2023). Thus, Uber continued to operate in compliance with existing regulations.

The struggle of taxis against new technologies is not only observed in the Uber debates, but like the Uber debates, it is also seen that a struggle is being developed against the applications of different technology platforms based in Türkiye that provide different transport services. Resistance in this area is not only focused on the urban use of traditional vehicles, but also on new modes such as the use of electric scooters or ride-sharing applications. Similarly, there have been long-running disputes between taxi drivers and “Martı,” a technology company / digital platform based in Türkiye which provides urban mobility, and the manager of Martı even claimed that he had been threatened by the president of ITEO (Birgün, 2022). In addition, a temporary injunction was issued following a request by the Istanbul Chamber of Taxi Drivers, and access to the ride-sharing applications of the relevant digital platform was blocked in connection with ‘pirate’ transport (Diken, 2023). On the context of all these developments, the functioning of digital platforms, which are space-centered and create urban mobility in Türkiye, offers an important area of discussion both in terms of labor and capital relations and as an area of intra-class competition. For this reason, it is necessary to evaluate these two different fields on the terms of their own characteristics.

## 4 Discussion

The influence of digital platforms on various occupations and employment relations demonstrates a range of impacts across different work domains, as well as the emergence of diverse divisions within the same occupational sphere. Regarding urban mobility, there are notable differences between platforms that provide delivery services and those that provide individual mobility, based on how labor is organized and examples of conflict in this area. The cause of this phenomenon is rooted in a comprehensive framework encompassing the structural features of labor organization to the institutionalization of specific services. In societies marked by late institutionalization - urbanization, the structural features lead to significant differences in the nature of the responses to platforms (OECD, 2023; ILO, 2018). The intangible nature of digital platforms is materialized on space, their activities at the regional level cause them to have a geopolitical nature, and the processes regarding the effectiveness of platforms are differentiated (Grohmann and Qiu, 2020). The position of platforms in capital accumulation processes leads to a debate on the effectiveness of local platforms in terms of becoming a regional power (Seto, 2024). When these features are evaluated together, platforms are positioned at a transitional point between the local, national and global spheres (Steinberg and Li, 2017). For example, the fact that the Türkiye-based “Armut” platform operates in more than one country under the name “HomeRun” (Uysal, 2023) is meaningful in terms of observing this transitivity. This can also be observed in the government’s approach to platform activities in Türkiye. The Investment Office of the Presidency of the Republic of Türkiye characterizes the acquisition of platforms in delivery services by global actors as a success story (The Investment Office of the Presidency of the Republic of Türkiye, 2023; The Investment Office of the Presidency of the Republic of Türkiye, 2024). Thus, the growth-oriented activities of delivery services have found a legitimate ground. In contrast, public authorities have taken a clear stance to protect local actors in the banning of Uber’s activities. This has contributed to the emergence of differences in the resistance and its outcomes in the two study areas.

Therefore, it is necessary to evaluate these two services in relation to platforms, different class positions involved in the process of institutionalization and capital accumulation. Moreover, while there are similarities in terms of urban mobility and labor utilization patterns of platforms providing delivery and transportation services, there are also important differences in the organization of platforms (Schmitd, 2017). The field of urban transportation does not only define a simple transportation service, but also points to an important public service. The state aims to maintain these services in a sustainable way through certain regulations (Çetin and Eryigit, 2011). This situation causes platforms providing services in urban transportation to operate in accordance with some existing regulations (Haidinger et al., 2024). The fact that Uber is organized to include only licensed taxis in line with existing regulations, as is the case with similar platforms, eliminates competition with local actors and thus the main problem area.

The mode of operation in delivery services, on the other hand, points directly to a private, interpersonal service provision and accordingly does not involve public service qualities or local government regulations regarding mobility. Workers encounter heightened risk during the hours designated as “rush hour” due to intensified labor ramifications in a structure where platforms control the labor process. The pressure of work in instant-demand delivery services is particularly challenging, creating significant risks relating to traffic accidents (Vecchio et al., 2022; Sarkies et al., 2022). However, many aspects such as income and social security are characterized by precariousness (Florisson and Mandl, 2018; Vieira, 2020; Popan and Anaya-Boig, 2021).

The developments emerging in working life on the basis of technological developments have created a new emphasis on the labor process (Thompson and Smith, 2009). This approach also refers to the differentiated employment relations observed on digital platforms (Omidi et al., 2023). Platforms have control over various elements, including revenue, time, and productivity. It is emphasized that the platforms’ control over labor and the labor process can be traced at three points. First, workers are constantly monitored by applications in the platform economy (Gandini, 2019; Veen et al., 2020). Second, the platforms create information asymmetries. Third and finally, the complex operational background of the applications makes it impossible for labor to develop various resistance mechanisms. Thus, the control of the labor process is shaped at the end of the labor process rather than during (Galière, 2020; Lin et al., 2020). Moreover, the deepening and diversifying possibilities of control make it possible to continuously reorganize labor according to the interests of the platforms (Van Doorn, 2020).

The problems surrounding employment status and working conditions have led to the emergence of new organizing movements and alternative models in this field. A comprehensive array of organized activities is witnessed, which extends from work slowdowns and solidarity-building processes to strikes, facilitated through social media and communication tools in tandem with state of the art technologies (Marrone and Finotto, 2019; Cant and Woodcock, 2021; Cini and Goldmann, 2021). It is also noted that for couriers, the physical connection they form with the urban environment plays a vital role in creating solidarity and a common class identity (Cini, 2023). Movements in this context are shaped outside the collective bargaining power of traditional trade unionism and, similar to nineteenth-century trade unionism (Joyce et al., 2023; Kresal, 2022) tend to focus on the formulation of legal regulations and involve regional movements spread across the city rather than workplace-based. This does not exclude the role of trade unions and their interventions in this area. It also shows that platform workers are in contact with existing trade unions. However, unions’ impact in this area varies regionally, historically and institutionally (Stuart et al., 2023). The couriers’ actions in Istanbul, which are against the platform’s regulations, also draw parallels with global literature. Demands to improve working conditions and reduce precariousness play a significant role. Moreover, new mechanisms of solidarity have been formed. There is a wide range of resistance to platform regulations, from informal organizations to joint struggles with formal unions. This situation bears resemblance to the history of trade union activities in the relevant sector in Türkiye, where there is a historically extensive repertoire of trade union activities in the transport sector (Kıdak, 2021; Makal, 2018).

Nevertheless, in Istanbul, the conflict between conventional taxi services and platform actors indicates that the issue is not solely a matter of labor relations. Rather, it points to a different field of struggle and to a set of structural problems and institutional arrangements that extend beyond the mere labor process. To gain a better comprehension of this phenomenon, it is important to outline briefly the impacts of rent relations in urban transport. The state imposes numerous regulations, especially restrictions on the supply of taxis, in order to maintain a balance between the quality of urban transport, which is a public service, and the income of those who provide it (Cooper and Mundy, 2016). Moreover, the free exchange of licenses in the market leads to significant rent potential, which depends on the class position, level of institutionalization and political activity of the service providers operating in this field. Therefore, depending on the regulations, small-scale providers of urban transport become a political pressure group that employs lobbying tactics to the extent of their institutionalization (Çetin and Eryigit, 2011).

Significant increases in potential license revenues are observed, especially due to supply constraints. The unavoidable outcome is occupational stratification in urban transportation through rent. Those previously engaged in urban transportation as small producers gradually evolve into employers or rentiers as a result of an increase in potential income. As a result, they are no longer employed in urban transportation, and a layered professional structure develops to the point where the values of urban transportation licenses are sold and rented (Khosa, 1994; Cervero and Golub, 2007; Seymour, 2009). In this context, Wyman (2013) describes the private property nature of urban transport licenses as “problematic private property,” pointing to the tense process of revenue generation. A similar property relation process is also visible in Istanbul. However, first, the role of rent on occupational stratification will be detailed.

Analyzing the relation between the phenomenon of “rent” and urban transport is crucial to understanding the struggle between different actors. Although rent refers to a set of relations that develops in relation to the creation of value on land (Marx, 1990), as Harvey (2016) emphasizes, rent debates generally emerge in a field where political economy encounters spatial organization and constitutes a means of claiming rights to potential income. Rentable property provides advantages for its owners, but it also deepens inequalities at the social level (Sørensen, 2005), thus contributes to the transformation of class positions (Harvey, 1985). Therefore, Katz (1986) draws attention to the fact that rent is a much more abstract conceptual phenomenon, as well as its capacity to deepen capitalist relations.

However, rent in urban transportation is highly fragile. Rent primarily benefits the first owner economically, but subsequent owners only receive the potential income. According to Sørensen (2005) and Holcombe (2018), transport licenses are capitalized based on projected revenue, thereby representing a direct investment opportunity. Therefore, license supplies can be reproduced by deregulation policies significantly faster than in the production of other commodities. The decline in potential income can lead to the fragmentation of rent-based stratification and class positions. The actors operating in this field have legitimized these restrictions through the discourse of investment protection and have resisted change and regulation in this area (Prentice et al., 2010; Wyman, 2013). The situation regarding the restrictions is also the case for Istanbul, and calculations based on the needs in Istanbul indicate that the number of taxis required in 2020 should be around 41,000 (Ergün et al., 2020).

Professional stratification is also central to the organization of traditional taxi services in Istanbul due to supply constraints. Furthermore, labor relations and relations with transport authorities are varied in Istanbul due to the absence of barriers to the circulation of transport licenses (Ersöz, 2022). This is also observable in the market value of licenses. In Istanbul, the price of taxi licenses has also risen in recent years, reaching up to 2 million TL (Gündoğdu, 2020), approx. $340,000 on the relevant date. In Istanbul, the market value of taxi licenses is determined by the potential revenue they can generate, with their rental value being as fragile as in other countries. As a matter of fact, the value of taxi licenses in Istanbul fell sharply when the local administration introduced a new taxi operating model for urban transport under its own authority (Sputniknews Türkiye, 2020). Although this implementation has not been put into practice, even the regulation attempt alone has a significant impact on license values in terms of observing the rent-generation process. Regarding this study, the change in the market value of taxi licenses as a result of Uber’s activities and the struggle mechanisms developed by traditional taxi license holders in this field are central. However, it is necessary to detail the differences arising from property relations.

The resistance against Uber’s activities points to a struggle that involves multiple layers. Although the struggle against Uber involves small producers holding urban transport licenses at the formal level and drivers at the informal level, it cannot be described as a class struggle. The resistance of traditional taxis against different platforms, especially Uber, which create a potential alternative through digital platforms, gains ground at two points. At first, traditional professional actors have aimed to prevent the impact of digital platforms in the formal sphere, both through their involvement in policy-making processes and through the support of policy makers. This is directly linked to the institutionalization of small producers who possess urban transport licenses. Indeed, as explained earlier, for small producers, competition processes carry the danger of dispossession and proletarianization. This is a significant reality in cases where taxi licenses define commodities with exchange values in the market. For example, in the case of New York, it is stated that the vast majority of taxis are not used by their license holders (Wyman, 2013). This process is also taking place in Istanbul, and a study in 2020 states that the vast majority of taxis in Istanbul are not used by their owners (Ergün et al., 2020). Moreover, Uber’s activities in Istanbul had a direct impact on drivers, and taxi drivers who started to operate in Uber emerged on the agenda. In particular, it is stated that Uber creates a bargaining space for the working conditions of drivers working with license holders and a regulatory effect in terms of labor relations (Ersöz, 2022). For urban transport license holders, the existence of digital platforms implies a significant loss of the value of their property, resulting in the loss of their current advantages and a shift in their social class position. In fact, the continuation of Uber’s activities with only taxi-licensed vehicles is an important example of the competitive axis of the current relation.

Furthermore, the conflict in this area has not only advanced through formal and bureaucratic means, but has also been encountered through informal measures on the field, occasionally involving violent actions. Although the struggle here involves the interventions of those working directly in the field, it is not possible to characterize that as a class struggle. As Thompson (1990) emphasizes, even if the class struggle does not involve a whole class, it should have a class character in terms of its aims. However, in this case, the informal actions are focused on preventing competition from the digital platforms, even if the actions are carried out by the drivers. The events do not involve a direct conflict between labor and capital. Rather, they represent a reflection of the intra-capital struggle between the existing small producers and the digital platform in the field. The tense relation between drivers and platforms is rooted in the complexity of the drivers’ class position. Although traditional taxi drivers in the field oppose digital platforms, their main goal is to preserve their employment opportunities by resisting the disappearance of small-scale production due to competition.

## 5 Conclusion

Evaluation of the platforms’ effectiveness in the context of Istanbul reveals diverse forms of labor resistance that are constantly mobile in the urban space. In terms of *delivery services*, the relations established with platforms create the reflections of a non-unionized laborization, precarity and informal organization for the working class that indicates emerging/developing class reflexes. However, in cases such as *taxi services* and Uber, where platform activity impacts the traditional organized structures of the professional field, the processes of struggle are replaced by political activity. For taxi license holders, who are already organized and demonstrate a petty bourgeois reflex in a profession stratified on the basis of rent, the relations and the networks of influence they have within the local and central government are the main determinant. In addition to the formal organization that interferes with public regulations, informal actions are also encountered, and struggles to prevent the fragmentation of potential income are organized in a way that may involve direct violence.

Resistance to platforms is visible in both occupational fields with varying levels of mobility in urban areas. However, the results differ according to their institutionalization. The struggle of the couriers against the platforms, although generally directed against the “independent contractor” organization of work, also reflects a general reflex related to the labor process. During the strikes, couriers achieved multiple benefits but also encountered harsh opposition from the platforms. Thus, demands for the legal regulation of labor platforms follow a bottom-up path.

On the other hand, actors organized as small businesses and operating directly within the local administration are able to take their struggles regarding the platforms directly to the political arena through their activities in political and bureaucratic field. As political pressure groups, actors within Istanbul’s traditional taxi services are effective in preventing or restricting the activities of the digital platforms. Thus, for these professions, the struggle within the platforms turns into a struggle for existence in terms of the preservation of their class positions. In this aspect, workers in urban transportation and delivery services represent “individual contractors” through small-scale production. However, when considered in terms of class positions, the reflexes of small producers against platforms in urban transport are at one end of the debate on the competition-based shaping of capital accumulation. The struggle of the actors in this field aims to prevent the decomposition of rent. Thus, their actions define a resistance to protect the current class positions. At other end of the debate labor in delivery services organizing against platforms represent a repetition of past experiences in a new digitalized playground as they fight to be recognized as “workers” and secure their rights. Consequently, a diverse spectrum of resistance is observed, encompassing both internal labor struggles within platforms and external opposition to platforms.

Finally, the development of platform labor in Türkiye presents a process shaped particularly in Istanbul. In this respect, the network of relations formed within the framework of the economic, political and therefore legal powers of the actors in the urban space is the fundamental basis for the rules of platform working relations in other cities. Comparative analyses of the examples in different urban spaces in Türkiye can help to follow the development of the process and also provide indicators for the future as well. The existence of new platforms and their impact will also constitute one of the important areas of research in the future. Considering the extent of working on platforms on a global scale and their increasing activities over the years, it is important to define the employment status in Türkiye in a way that includes new categories in accordance with the global field and to implement regulations to secure labor rights. Moreover, the multidimensional characteristics of the platforms’ organization require the consideration of different stakeholders in these regulations.

## Footnotes

^1^As a matter of fact, in a related issue to this study, the new taxi system project of the local government has been rejected many times with the opposing votes of the representatives of the profession and the central government officials. This situation constituted an important example of the tripartite and tense structure between the central government, taxi drivers and local government (Altınoklu, 2023).
